# Association of VCS2000-Measured Carcass Traits with Anatomical Site-Specific Fat Deposition and Appearance Acceptability of Pork Belly

**DOI:** 10.3390/ani16203242

**Published:** 2026-10-15

**Authors:** Yeongkwon Yun, Boin Lee, Young Min Choi

**Affiliations:** 1 Department of Animal Sciences, Kyungpook National University, Sangju-si 37224, Republic of Korea; cp356@ekape.or.kr; 2 Korea Institute for Animal Products Quality Evaluation, Sejong-si 30100, Republic of Korea; 3 Division of Food Technology and Nutrition, Sunmoon University, Asan-si 31460, Republic of Korea; ananassab9320@gmail.com

**Keywords:** carcass traits, VCS2000, pork belly, fat accumulation

## Abstract

The primary objective of this study was to evaluate the utility of automated carcass parameters (VCS2000) in reflecting site-specific fat distribution along the thoracic (TV) and lumbar vertebrae (LV) of pork bellies. Additionally, this study aimed to determine how VCS2000-derived carcass weight and fat proportion influence localized fat accumulation and the resulting visual acceptability of these distinct anatomical sites. Total fat content, evaluated via the VCS2000 system, showed a positive loading on principal component 1 along with carcass weight, backfat thickness at various sites, and fat percentages at anatomical belly sites, demonstrating a close association with specific belly traits. Considering the carcass weight, belly weight, and belly fat accumulation at anatomical belly sites, carcasses with a higher weight but a lower fat percentage exhibited more favorable belly traits among all evaluated carcasses (*p* < 0.05). Anatomical location significantly influenced visual acceptability, with panelists preferring leaner sections (the 13th and 14th TV) over those with heavy fat accumulation (the 10th and 11th TV; *p* < 0.05). These results suggest that visual acceptability may be primarily driven by the localized distribution and structural balance of fat and lean meat, rather than the absolute fat proportion of pork belly.

## Simple Summary

Pork belly is highly valued globally, but excessive or unevenly distributed fat in belly slices often negatively affects consumer acceptance and marketability. The challenge for the meat industry is that traditional grading methods cannot easily detect how fat is unevenly distributed within specific parts of the belly. Therefore, this study explored whether a digital imaging system (VCS2000) could explain belly fat distribution and investigated what specific visual traits drive consumer choices. Our findings revealed that the VCS2000 measurements were closely associated with belly quality. Furthermore, sensory evaluation showed that trained panelists were sensitive to the physical structure of the meat; they strongly favored sections with a clean, alternating pattern of lean meat and fat, while rejecting cuts with heavy fat accumulation. These results demonstrate that localized fat distribution is more critical than the total amount of fat. Ultimately, these insights may inform meat sorting strategies to better align with consumer demands, while their potential application in swine breeding programs requires further genetic evaluation.

## 1. Introduction

Pork continues to account for the largest proportion of total global meat consumption, particularly in countries such as China, Germany, and South Korea, despite the growing preference for poultry meat [1,2]. Thus, the swine industry has heavily prioritized maternal and carcass production traits, such as litter size and lean meat yield, by employing optimized selection and feeding systems [3,4]. To accommodate the expanding slaughter volume, domestic slaughterhouses have recently implemented automated equipment to standardize and streamline the grading process traditionally performed by trained graders [1,5]. Among these automatic carcass grading devices, the VCS2000 (e+v Technology GmbH, Oranienburg, Brandenburg, Germany) utilizes a vision-based image analyzer as a digitalized evaluation system [6]. This provides real-time, non-destructive data on carcass traits, including carcass weight, carcass composition, backfat thickness, and primal cut weights, thereby mitigating the potential variations associated with subjective visual assessments [1].

While pork loin and ham are highly valued cuts in many Western markets, the belly represents one of the most preferred cuts in South Korea, positioning it as the most economically significant premium cut in the domestic market [2,7]. Generally, pork belly exhibits a higher fat content compared to other cuts, and this fat level is closely linked to the organoleptic characteristics of cooked pork, including tenderness, juiciness, and flavor, which consequently strongly influence consumer purchase intention [5,8]. Within this market, where pork belly is commonly retailed and consumed as sliced meat, consumers can readily perceive the relative proportions and distribution of lean and adipose tissues, making visible fat an important attribute in purchase decisions [2]. Korean consumers generally prefer pork belly with a moderate proportion of fat, whereas excessive or localized fat accumulation may influence the visual characteristics of retail-ready belly slices [2,7]. Moreover, excessive fat may be perceived as undesirable by health-conscious consumers because of the reported association between high fat consumption and chronic diseases, such as obesity and hypertension [9]. Therefore, both the amount and visible distribution of fat are important considerations for the consumer acceptance and marketability of pork belly, prompting producers to minimize excessive or localized fat aggregates [7].

The morphological and compositional characteristics of pork belly are influenced by both genetic and environmental factors [10]. Generally, slaughter weight and backfat thickness, which are integral components of carcass quality assessments, exhibit a positive correlation with the fat content of pork belly; consequently, these carcass traits have been utilized to estimate not only overall carcass yield but also pork belly composition [10,11]. However, these carcass traits may not fully reflect the detailed composition of pork belly, which comprises various muscles and intermuscular fat that vary significantly depending on the specific anatomical locations across the thoracic (TV) and lumbar vertebrae (LV) [12]. Thus, assessment of site-specific fat characteristics across the belly is necessary to better characterize its anatomical variation and provide relevant information for producers, suppliers, and consumers. While previous studies primarily focused on overall carcass grading, this study specifically investigates whether automated whole-carcass measurements derived from the VCS2000 system can explain localized, site-specific fat distribution within the pork belly and evaluate its association with visually preferred characteristics. Therefore, this study aimed to investigate the relationship between carcass traits measured by the VCS2000 and locational fat traits of pork belly across the TV and LV regions. In addition, the effects of VCS2000-derived carcass weight and fat percentage on the visual characteristics of pork belly slices, including the degree of fat accumulation and appearance acceptability, were examined to provide information relevant to pork belly production and quality management.

## 2. Materials and Methods

### 2.1. Animals

This study used data provided by the Korea Institute of Animal Products Quality Evaluation (KAPE) from 100 commercial pigs (Landrace × Yorkshire × Duroc; 54 barrows and 46 gilts). The pigs were slaughtered following the standard procedures outlined by the KAPE [13] over three consecutive days at the same abattoir, with 30 to 35 pigs processed per day, and the carcasses were graded by trained evaluators. Information on animal age and detailed rearing conditions, including diet and housing, was not available in the provided dataset; therefore, their potential effects on carcass and belly characteristics could not be evaluated in this study. Data obtained from KAPE included hot-carcass weight, marbling score assessed on a scale from 1 to 5 (low to high marbling) on the loin muscle surface between the last TV and first LV, intermuscular fat thickness (IFT) between the *latissimus dorsi* and *cutaneous trunci* muscles (at the 4th–5th thoracic vertebra cross-section), and backfat thickness (BT). BT measurements included the mean value from two standard grading sites (the last rib to first LV and the 11th to 12th ribs), as well as specific points at the 4th, 12th, 13th, and 14th TV and the 7th LV.

The measurements parameters and prediction accuracy of the VCS2000 were configured in accordance with the established settings from previous literature [1,6,14]. By applying image-processing algorithms to half-carcass scans, the automated system generated data on carcass weight (VCW), total lean meat (VLW) and fat weights (VFW), lean meat (VLP) and fat percentages (VFP), and detailed belly traits, including belly weight (VBW), belly fat weight (VBFW), and belly fat percentage (VBFP).

### 2.2. Image Analysis for Pork Belly

Following a 24 h chilling at 4 °C, a total of 100 pork belly samples were collected from the left half of each carcass in accordance with the standard cutting guideline of KAPE [13]. Vertical cross-sections of each belly were then prepared from the dorsal to the ventral side at the specific anatomical sites of the 6th, 10th, 11th, 12th, 13th, and 14th TV, and the 4th LV. To acquire hyperspectral data, belly slices obtained from each carcass were scanned in reflectance mode using a Cubert Ultris X20 Plus hyperspectral camera (Cubert, Ulm, Baden-Württemberg, Germany) under halogen illumination. Image capturing and data collection were carried out through the CUVIS operating software (Cubert). To quantify the respective volumes of fat tissue within belly slices, the perClass Mira Software package (version 5.4; Delft, Zuid-Holland, Netherlands) was utilized, following the methodology described in a previous study [15].

### 2.3. Sensory Evaluation

A total of 700 pork belly slices (seven distinct anatomical locations × one hundred bellies) were imaged using the Cubert Ultris X20 Plus hyperspectral camera. The high-resolution digital images (JPEG format, 3024 × 4032 pixels) were captured under standardized lighting conditions. Sensory evaluations were conducted by a panel of ten trained individuals (six women and four men aged 23–48 years) who had completed at least 6 months of specialized training at the Muscle Biology Laboratory of Kyungpook National University (KNU 2019–0027), following the standard protocols outlined by the American Meat Science Association [16]. For the evaluation, each sample was coded with a three-digit number. The assessment was performed across 90 individual sessions, with 6 to 8 samples presented per session. During each session, the standardized images were displayed to the panelists via a 40-inch computer monitor for evaluation. Panelists evaluated the belly samples using a 9-point hedonic scale to assess the degree of fat accumulation (1 = not at all, 9 = extremely high) and overall appearance acceptability (1 = dislike extremely, 9 = like extremely).

### 2.4. Statistical Analysis

The individual carcass was considered the experimental unit. To evaluate differences in carcass traits between castrated male and female pigs, data were analyzed using the general linear model (GLM) procedure, with sex included as a fixed effect. Significant differences between the least squares mean (LSM) of the two groups were determined using the PDIFF option at *p* < 0.05, and results were expressed as LSM with standard errors (SE). Principal component analysis (PCA) based on a correlation matrix was performed using the PRINCOMP procedure in SAS software (version 9.4; SAS Institute, Cary, NC, USA) to assess the multivariate relationships among grader-evaluated carcass traits, VCS2000-derived parameters, and fat percentages determined by image analysis at different anatomical sites of the belly. PCA was used to reduce the dimensionality of correlated variables and identify the primary carcass and belly characteristics associated with fat accumulation in different vertical cross-sections of the belly.

Carcass weight and fat percentage measured by the VCS2000 were clustered separately using the FASTCLUS procedure in SAS. Three clusters were specified for carcass weight, resulting in heavy (H; 90.4–102.3 kg; *n* = 34), medium (M; 84.3–90.1 kg; *n* = 39), and light (L; 76.6–84.1 kg; *n* = 27) weight groups. Two clusters were specified for carcass fat percentage, resulting in high-fat (H; 29.1–42.0%; *n* = 60) and low-fat (L; 18.1–28.9%; *n* = 40) groups. Initial cluster seeds were selected from the observations by the FASTCLUS procedure, and observations were assigned to clusters based on their proximity to the cluster centers. The resulting group boundaries were specific to the present sample and were used within-study comparisons rather than as industry grading thresholds. The resulting six interactive combinations comprised the following groups: HH (*n* = 26), HL (*n* = 8), MH (*n* = 24), ML (*n* = 15), LH (*n* = 10), and LL (*n* = 17). To evaluate the main and interaction effects of carcass weight and fat percentage classes on carcass traits, the GLM procedure was used, with carcass weight class, fat percentage class, and their interaction included as fixed effects. For belly characteristics measured at seven anatomical locations within the same carcass, repeated-measures analysis was performed using a linear mixed model (MIXED procedure). In this model, carcass weight class, fat percentage class, anatomical location, and their interactions were included as fixed effects, while the individual carcass was specified as the subject for repeated measurements, and daily batch was included as a random effect. For sensory assessments, the MIXED procedure was employed, with carcass weight class, fat percentage class, and their interaction included as fixed effects and panelist as a random effect. Significant differences among the main effects and their combinations were determined using the PDIFF option at *p* < 0.05, and data were presented as LSM with SE.

## 3. Results

### 3.1. Carcass Traits of Castrated Male and Female Pigs

Table 1 compares carcass traits (assessed via visual grading by trained evaluators and automated instrumentation by VCS2000) and belly fat percentage determined by image analysis between castrated male and female pigs. There were no significant differences in carcass traits between the sexes (*p* > 0.05), with the exception of the total belly fat percentage obtained by the VCS2000 system. Total belly fat percentage was significantly lower in female pigs compared to castrated male pigs (33.6 vs. 35.8%, *p* = 0.037), although no differences were detected in the belly fat percentages at the specific anatomical sites (6th–14th TV and 4th LV) (*p* > 0.05). 

### 3.2. Relationships Between Carcass Traits and Belly Characteristics

**Figure 1:**
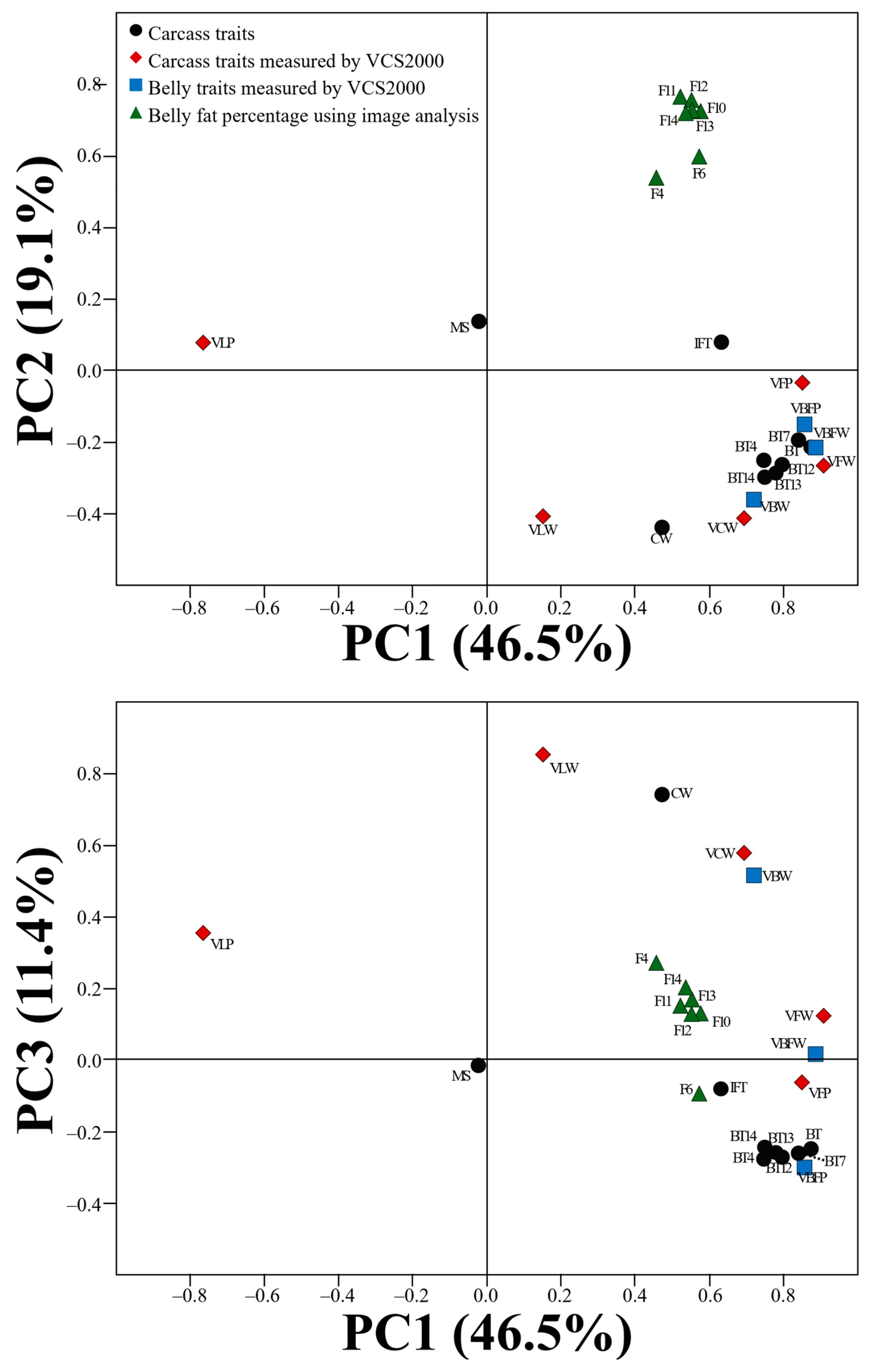
Principal component analysis (PCA) plots for PC1 versus PC2 and PC1 versus PC3. The percentage variance explained by the three PCs was 77.0%. CW, carcass weight; BT, grading backfat thickness; MS, marbling score; VCW, carcass weight measured by VCS2000; VLW, total lean meat weight measured by VCS2000; VLP, total lean meat percentage measured by VCS2000; VFW, total fat weight measured by VCS2000; VFP, total fat percentage measured by VCS2000; VBW, belly weight measured by VCS2000; VBFW, belly fat weight measured by VCS2000; VBFP, belly fat percentage measured by VCS2000; F6, belly fat percentage at 6th thoracic vertebra (TV) using image analysis; F10, belly fat percentage at 10th TV using image analysis; F11, belly fat percentage at 11th TV using image analysis; F12, belly fat percentage at 12th TV using image analysis; F13, belly fat percentage at 13th TV using image analysis; F14, belly fat percentage at 14th TV using image analysis; F4, belly fat percentage at 4th lumbar vertebra (LV) using image analysis; BT4, backfat thickness at 4th TV; BT12, backfat thickness at 12th TV; BT13, backfat thickness at 13th TV; BT14, backfat thickness at 14th TV; BT7, backfat thickness at 7th LV; IFT, intermuscular fat thickness between the *latissimus dorsi* and *cutaneous trunci* muscles (at the 4th–5th TV cross-section).

To evaluate relationships among variables, PCA was conducted to reduce data dimensionality across visually graded carcass traits, VCS2000 instrumentation data, and fat percentages at anatomical belly sites (Figure 1). Across the 24 variables, the first three principal components (PCs) accounted for approximately 77.0% of the overall variance, with PC1, PC2, and PC3 contributing 46.5, 19.1, and 11.4%, respectively. It should be noted that several VCS2000-derived variables are algebraically related, and their simultaneous inclusion may therefore have increased the contribution of the underlying carcass weight- and fat-related variation to component structure. Carcass weight and fat percentages at each belly site were located in the positive side along PC1, reflecting a positive correlation among them. BT at standard grading sites, VCW, VFP, and VFW were likewise positioned on the positive side of PC1. VLP showed a negative loading on PC1, whereas IFT showed a positive loading, indicating an inverse relationship with these variables. Meanwhile, carcass weight, VCW, VLW, and VBW exhibited positive loadings on PC2, and carcass weight and VBFP were characterized by negative loadings on PC3.

### 3.3. Carcass Characteristics Across Groups Categorized by VCS2000 Carcass Weight and Fat Percentage

Various carcass traits evaluated by trained assessors and automated instrumentation across groups categorized by VCS2000 carcass weight and fat percentage are presented in Table 2. No significant interactions between carcass weight and fat percentage classes were observed for the carcass traits measured by evaluators and the VCS2000 system (*p* > 0.05). The heavy group exhibited a significantly higher carcass weight compared to the medium and light groups (93.9 vs. 89.0 and 84.3 kg, *p* < 0.001). No significant differences were observed in marbling score among the groups (*p* > 0.05). In contrast, BT differed significantly among the groups (*p* < 0.05), being thicker in the heavy group compared to the medium and light groups (24.5 vs. 22.4 and 21.4 mm, *p* = 0.003), whereas no difference was observed between the medium and light groups (*p* > 0.05). Within the heavy group, there were no differences in BT from the 4th to 14th TV between the high- and low-fat groups; however, BT at the 7th LV was significantly thicker in the HH group than in the HL group (20.1 vs. 16.1 mm, *p* < 0.001). Moreover, the LH group had a significantly thicker BT at 7th LV compared to the LL group (17.3 vs. 13.6 mm, *p* < 0.001). The ML and LL groups exhibited lower IFT compared to the HH group (7.31 and 8.16 vs. 10.1 mm, *p* = 0.017). 

Similar to the trend observed in carcass weight, VCW differed significantly among the heavy, medium, and light groups (93.5, 87.2, and 81.6 kg, respectively, *p* < 0.001), although no difference was observed between the high- and low-fat groups within each carcass weight group (*p* > 0.05). The HL group exhibited the highest VLW among all groups (60.2 kg, *p* < 0.001), whereas no difference was detected between the HH and ML groups (57.8 vs. 57.7 kg, *p* > 0.05). Within each carcass weight group, the high-fat group had a significantly higher VFW than the low-fat group (*p* < 0.001). VLP was higher in the low-fat group than in the high-fat group (*p* < 0.001), whereas VFP exhibited the opposite pattern, being higher in the high-fat group than in the low-fat group within each carcass weight group (*p* < 0.001). Additionally, the HH group had a higher VFP compared to the MH and LH groups (34.4 vs. 31.9 and 31.2%, *p* < 0.001), whereas no significant difference was observed among the HL, ML, and LL groups (27.5, 26.2, and 25.7%, respectively, *p* > 0.05).

### 3.4. Pork Belly Characteristics Across Groups Categorized by VCS2000 Carcass Weight and Fat Percentage

Table 3 compares belly characteristics assessed by VCS2000 and image analysis across groups categorized by carcass weight and fat percentage. No significant interactions between carcass weight and fat percentage classes were observed for VCS2000-meausred belly characteristics or belly fat percentages at the evaluated anatomical locations (*p* > 0.05). The heavy group had a higher VBW compared to the medium and light weight groups (13.1 vs. 12.2 and 11.5 kg, respectively, *p* < 0.001). Although the main effect of VCS2000-based fat percentage class on VBW was significant (*p* < 0.001), pairwise comparisons between the high- and low-fat groups within each carcass weight group revealed no significant differences (*p* > 0.05). In contrast, the high-fat group exhibited greater VBFW and VBFP compared to the low-fat group within each carcass weight group (*p* < 0.001), whereas a significant difference was observed in VBFW among the heavy, medium, and light groups (4.63, 4.08, and 3.64 kg, respectively, *p* < 0.001). 

Regarding belly fat percentage, the main effect of carcass weight class was not significant at any of the evaluated anatomical locations (*p* > 0.05), whereas the effect of fat percentage class was significant at most locations (*p* < 0.05). No differences were observed among the HH, MH, and LH groups across the evaluated anatomical locations (6th–14th TV and 4th LV; *p* > 0.05). The HH group had a higher fat percentage at the 13th TV (37.9 vs. 32.8%, *p* = 0.002) and 14th (35.6 vs. 30.4%, *p* < 0.001) compared to the HL group, although no difference was observed between the two groups at the other anatomical sites (*p* > 0.05). Within the medium group, the ML group exhibited a lower percentage across most anatomical sites compared to the MH group (*p* < 0.05), except for the 10th TV (40.9 vs. 45.3%, *p* > 0.05) and 4th LV (31.1 vs. 33.7%, *p* > 0.05). Within each group, significant location-dependent differences in belly fat proportion were observed (*p* < 0.001), where the fat percentages at the 10th and 11th TV were generally higher compared to the other anatomical sites (*p* < 0.05), although the difference was not statistically significant in certain groups (*p* > 0.05).

### 3.5. Fat Deposition and Visual Acceptability of Belly Cuts Across Groups Categorized by VCS2000 Carcass Weight and Fat Percentage

Table 4 presents the visual assessment of fat accumulation and appearance acceptability evaluated by trained panelists for each group. Significant interactions between carcass weight and fat percentage classes were observed for the degree of fat accumulation at the 6th, 10th, and 13th TV and the 4th LV (*p* < 0.05). Across all anatomical sites of pork belly, the HH group exhibited higher fat accumulation scores compared to the other groups (*p* < 0.05), with the exception of the 12th TV, where no significant difference was found between the HH and LH groups (3.65 vs. 3.38, *p* > 0.05). No significant differences were observed between the HL and LL groups at any belly slice position (*p* > 0.05). Moreover, with the exception of the 4th LV (1.98 vs. 1.64, *p* < 0.001), there were no differences between the MH and LH groups at any position (*p* > 0.05). Similar to the fat percentage measured by image analysis, significant location-dependent differences in the degree of fat accumulation were observed within each group (*p* < 0.001). Fat accumulation was significantly higher at the 10th TV than at the other pork belly sites across all groups (*p* < 0.001), except for the comparison with the 11th TV in the LL group (*p* > 0.05). The 14th TV and the 4th LV exhibited lower fat accumulation than the other sites within the HH or MH groups (*p* < 0.001).

In terms of appearance acceptability, significant interactions between two classes were observed at all anatomical locations (*p* > 0.05) except the 12th TV (*p* = 0.057). The HH group exhibited lower acceptability scores than the HL group across all belly slices (*p* < 0.001). Additionally, this group showed lower scores compared to the MH and LH groups at all evaluated points (*p* < 0.05), with the exception of the 12th TV, where no significant differences were found among the HH, MH, and LH groups (5.69, 6.11, and 6.05, respectively, *p* > 0.05). In contrast, the LH group had higher scores compared to the LL group at the 6th TV (7.10 vs. 6.51, *p* < 0.05), 10th TV (5.38 vs. 4.79, *p* < 0.05), and 4th LV (6.50 vs. 5.82, *p* < 0.05), although no differences were observed at the other sites (*p* > 0.05). Furthermore, appearance acceptability also varied significantly across the sampling locations (*p* < 0.001). Specifically, the visual acceptability at the 10th TV was markedly lower than those at the 12th, 13th, and 14th TV within each group (*p* < 0.001). Moreover, the visual acceptability of the 6th TV was higher than that at the 4th LV within each group (*p* < 0.05), except for the HH group, where no difference was observed (6.16 vs. 5.87, *p* > 0.05).

## 4. Discussion

Porcine live and carcass traits are strongly affected by a complex interplay of genetic and environmental factors [17,18]. Among these factors, sex has been reported to significantly affect carcass quality characteristics [2,15]. Castration alters the hormonal profile; thus, barrows tend to exhibit faster growth rates, which result in heavier carcass weights at a given age and greater fat deposition compared to intact boars and gilts [2]. However, while some studies have reported differences in BT between barrows and gilts [2,19], carcass weights at a similar age did not always differ between the sexes [2]. Additionally, Jo et al. [15] noted no significant differences in BT and belly fat content at specific anatomical sites, but rather observed that gilts yielded a heavier carcass weight than barrows (*p* < 0.05). Consistent with these varying results, in the present study, no significant effects of sex were observed for the carcass traits assessed by trained evaluators and VCS2000, nor for the belly fat percentages across the measured sites (*p* > 0.05). However, an exception was noted in the overall fat percentage of pork belly; barrows had a higher fat percentage than gilts (*p* < 0.05) when assessed by the VCS2000 system.

In general, strong correlations were observed among the manually assessed carcass traits [5]. Carcass weight correlated positively with the weights of major primal cuts (including pork belly, ham, loin, and picnic shoulder), but showed little to no association with fat measurements [5]. Conversely, BT is one of the key parameters used to estimate carcass quality grading, and it showed a significant association with the proportion of lean meat and fat in the carcass and individual cuts [5,15]. Thus, while these parameters are useful for overall carcass grading, they may not adequately reflect localized fat variation in pork belly, which has been reported to influence consumer preference and purchase intention. Indeed, Jo et al. [15] found no significant correlation between carcass traits (including carcass weight and BT) and fat percentages at specific belly sites. In the present study, the VCS2000-derived fat percentage showed positive loading on PC1, along with BT measured at various sites and fat percentages at anatomical belly sites. Additionally, VFP was positively correlated with both the marbling score measured on the cut surface of the loin muscle between the last TV and first LV and IFT between the *latissimus dorsi* and *cutaneous trunci* muscles. Overall, both VFP and VCW were associated with belly characteristics, including belly weight and fat percentage. These findings align with the previous result that the VCS2000 is useful for evaluating porcine carcass traits based on image analysis [20]. Therefore, in this study, the porcine carcasses were clustered into distinct classes based on these parameters to evaluate the differences in specific belly characteristics across the groups, including fat percentage, degree of fat deposition, and visual acceptability.

The primary objective of swine breeding programs has consistently been the enhancement of productivity, and conventional parameters used to define these traits encompass growth rate, loin eye area (LEA), and BT as primary indicators of skeletal muscle development [7]. To achieve this objective, commercial producers strategically prioritize these economically important traits, as they exhibit high heritability and respond efficiently to selection [7]. Under the United States Department of Agriculture standards, variables such as carcass weight, along with 10th-rib LEA and BT, have been utilized to estimate the proportion of lean and fat in porcine carcasses [21]. In the present study, BT was evaluated across multiple locations to assess its relationship with total fat proportion and carcass weight. Notably, little to no association was found regarding fat content; carcasses possessing higher fat percentages did not consistently present with thicker BT than leaner carcasses. Moreover, the extent of intramuscular fat on the loin cross-section was independent of both carcass weight and overall fat proportion. However, VFW showed an overall significant decrease from the HH to LL groups (HH > HL = MH > ML = LH > LL, *p* < 0.05), and the high-fat group exhibited a lower VLP than the low-fat group within each carcass weight group (*p* < 0.05). Taken together, these findings suggest that carcass weight and total fat content, as measured by the VCS2000 system, can serve as reliable determinants for elucidating overall carcass characteristics, as well as pork belly traits. The lack of significant interactions between carcass weight and fat percentage classes for most evaluated traits suggests that the effect of overall carcass fatness on localized belly fat distribution operates independently of carcass weight.

While pork belly fat comprises subcutaneous, intermuscular, and intramuscular fat, this study placed a special emphasis on evaluating the visible macroscopic layers, including subcutaneous and intermuscular fat. Regarding the degree of fat accumulation, the HH group exhibited higher levels across the seven anatomical belly sites compared to the other groups (*p* < 0.05), which correspondingly resulted in lower visual acceptability across most of these regions compared to the HL, MH, and ML groups. In contrast, visual acceptability was generally comparable between the HL and MH groups (*p* > 0.05), except at the 11th and 12th TV (*p* < 0.05). Similarly, differences between the HL and ML groups were not statistically significant at most belly regions (*p* > 0.05), except at the 6th TV and 4th LV (*p* < 0.05). In the South Korean market, the attractiveness of pork belly is generally determined by visual and structural parameters, including fat content, belly width, meat color, and fat color. Among these parameters, fat content is the most critical factor driving consumer preference [22,23]. In the present study, with assessed visual acceptability using trained panelists, excessive fat clumping in belly sites (such as the 10th and 11th TV) resulted in lower acceptability compared to those with lower fat accumulation (such as the 13th and 14th TV). However, despite having lower fat accumulation, the 4th LV site yielded a lower acceptability score compared to other anatomical sections within each group. This lower preference may be primarily attributed to its structural limitation, specifically the lack of characteristic alternating layers of fat and lean meat. Thus, the present findings suggest that site-specific fat distribution plays a critical role in determining the visual acceptability of pork belly slices.

## 5. Conclusions

This study demonstrates that carcass weight and total fat content evaluated via the VCS2000 system are associated with overall carcass characteristics and specific pork belly traits. When analyzing carcass weight, belly weight, and belly fat accumulation at anatomical belly sites, carcasses exhibiting a higher weight combined with a lower fat percentage presented more favorable belly traits among all evaluated carcasses. Furthermore, anatomical location significantly influenced visual acceptability; panelists tended to assign higher acceptability scores to leaner sections (the 13th and 14th TV) compared to sections showing higher fat accumulation (the 10th and 11th TV). Ultimately, these results indicate that the visual acceptability of pork belly is influenced not simply by its overall fat proportion but by the localized distribution of fat and its relative balance with lean tissue.

## Figures and Tables

**Table 1:** Comparison of carcass traits between barrows and gilts.

	Barrows (*n* = 54)	Gilts (*n* = 46)	*p*-Value
***Carcass measurements***			
Carcass weight (kg)	88.6 (0.57) ^1^	90.0 (0.63)	0.225
Grading backfat thickness (BT; mm) ^2^	23.4 (0.50)	22.4 (0.55)	0.099
Intermuscular fat thickness (mm) ^3^	9.61 (0.40)	8.28 (0.43)	0.052
Marbling score	2.86 (0.16)	2.67 (0.17)	0.124
***VCS2000 measurements***			
Carcass weight (kg)	87.4 (0.72)	88.5 (0.79)	0.214
Total lean meat weight (kg)	47.7 (0.37)	49.4 (0.40)	0.063
Lean meat percentage (%)	54.6 (0.48)	55.8 (0.53)	0.443
Total fat weight (kg)	26.5 (0.54)	26.6 (0.60)	0.821
Fat percentage (%)	30.3 (0.57)	30.1 (0.62)	0.895
Belly weight (kg)	12.2 (0.12)	12.4 (0.13)	0.364
Fat weight of belly (kg)	4.32 (0.11)	4.13 (0.12)	0.633
Fat percentage of belly (%)	35.8 ^a^ (0.57)	33.6 ^b^ (0.62)	0.037
***Fat percentages of belly cuts using image analysis***			
Fat percentage at 6th thoracic vertebra (%)	35.5 (0.79)	34.9 (0.84)	0.767
Fat percentage at 10th thoracic vertebra (%)	43.3 (0.89)	43.1 (0.97)	0.782
Fat percentage at 11th thoracic vertebra (%)	40.0 (0.93)	40.9 (1.02)	0.302
Fat percentage at 12th thoracic vertebra (%)	37.8 (0.99)	38.2 (1.08)	0.586
Fat percentage at 13th thoracic vertebra (%)	36.3 (0.93)	36.5 (1.02)	0.670
Fat percentage at 14th thoracic vertebra (%)	33.7 (0.83)	34.5 (0.92)	0.281
Fat percentage at 4th lumbar vertebra (%)	31.0 (0.91)	31.9 (0.99)	0.524

Data was analyzed using a general linear model. ^a,b^ Different superscripts in the same row represent significant differences (*p* < 0.05); ^1^ standard error of least-square means; ^2^ grading backfat thickness: the average of two grading sites (BT between the last rib and first lumbar vertebra; BT between the 11th and 12th ribs); ^3^ intermuscular fat thickness: measured between the *latissimus dorsi* and *cutaneous trunci* muscles (at the 4th–5th thoracic vertebra cross-section).

**Table 2:** Porcine carcass traits in groups categorized by VCS2000-measured weight and fat percentage.

Carcass Weight Measured by VCS2000 (W)	Heavy		Medium		Light		*p*-Value		
Fat Percentage Measured by VCS2000 (F)	High (*n* = 26)	Low (*n* = 8)	High (*n* = 24)	Low (*n* = 15)	High (*n* = 10)	Low (*n* = 17)	W	F	W × F
***Carcass measurements by evaluators***									
Carcass weight (kg)	93.1 ^a^ (0.45) ^1^	94.6 ^a^ (0.51)	88.3 ^b^ (0.47)	89.8 ^b^ (0.59)	83.8 ^c^ (0.73)	84.6 ^c^ (0.56)	<0.001	0.010	0.768
Marbling score	2.61 (0.26)	3.00 (0.43)	2.92 (0.24)	2.86 (0.27)	2.56 (0.38)	2.64 (0.27)	0.530	0.464	0.715
Grading backfat thickness (BT; mm) ^2^	25.7 ^a^ (0.63)	23.6 ^ab^ (1.13)	23.0 ^b^ (0.65)	21.3 ^bc^ (0.82)	23.7 ^ab^ (1.01)	19.6 ^c^ (0.77)	0.003	<0.001	0.474
BT at 4th thoracic vertebra (mm)	34.7 ^a^ (0.84)	31.6 ^ab^ (1.53)	31.0 ^b^ (0.88)	30.7 ^b^ (1.11)	31.3 ^ab^ (1.34)	28.4 ^b^ (1.05)	0.009	0.003	0.444
BT at 12th thoracic vertebra (mm)	26.4 ^a^ (1.29)	24.9 ^ab^ (2.32)	23.9 ^ab^ (1.34)	22.5 ^bc^ (1.69)	24.4 ^ab^ (2.08)	20.8 ^c^ (1.59)	<0.001	0.129	0.924
BT at 13th thoracic vertebra (mm)	25.0 ^ab^ (0.84)	27.1 ^a^ (1.52)	23.0 ^bc^ (0.88)	21.6 ^bc^ (1.11)	23.1 ^bc^ (1.36)	19.6 ^c^ (1.04)	<0.001	0.218	0.079
BT at 14th thoracic vertebra (mm)	24.6 ^a^ (0.59)	23.0 ^ab^ (1.06)	22.9 ^ab^ (0.61)	21.9 ^bc^ (0.78)	22.7 ^ab^ (0.95)	19.7 ^c^ (0.73)	0.009	<0.001	0.725
BT at 7th lumbar vertebra (mm)	20.1 ^a^ (0.68)	16.1 ^bc^ (1.22)	17.2 ^b^ (0.71)	15.7 ^bc^ (0.89)	17.3 ^b^ (1.11)	13.6 ^c^ (0.85)	0.012	<0.001	0.524
Intermuscular fat thickness (mm) ^3^	10.1 ^a^ (0.70)	8.50 ^ab^ (1.12)	8.72 ^ab^ (0.64)	7.31 ^b^ (0.71)	8.21 ^ab^ (0.99)	8.16 ^b^ (0.70)	0.893	0.017	0.491
***Carcass measurements by VCS2000***									
Carcass weight (kg)	94.1 ^a^ (0.42)	92.9 ^a^ (0.77)	87.6 ^b^ (0.44)	86.9 ^b^ (0.56)	82.3 ^c^ (0.69)	80.9 ^c^ (0.53)	<0.001	0.028	0.782
Total lean meat weight (kg)	57.8 ^b^ (0.36)	60.2 ^a^ (0.65)	55.7 ^c^ (0.38)	57.7 ^b^ (0.48)	52.6 ^e^ (0.59)	54.3 ^d^ (0.45)	<0.001	<0.001	0.679
Total fat weight (kg)	30.3 ^a^ (0.43)	26.3 ^b^ (0.77)	26.0 ^b^ (0.44)	23.1 ^c^ (0.56)	24.0 ^c^ (0.69)	20.5 ^d^ (0.53)	<0.001	<0.001	0.581
Lean meat percentage (%)	52.4 ^c^ (0.56)	57.5 ^a^ (1.01)	54.0 ^b^ (0.59)	57.9 ^a^ (0.74)	54.0 ^bc^ (0.90)	57.9 ^a^ (0.69)	0.396	<0.001	0.598
Fat percentage (%)	34.4 ^a^ (0.48)	27.5 ^c^ (0.86)	31.9 ^b^ (0.49)	26.2 ^c^ (0.63)	31.2 ^b^ (0.77)	25.7 ^c^ (0.59)	<0.001	<0.001	0.486

^a–e^ Different superscripts in the same row represent significant differences (*p* < 0.05); ^1^ standard error of least-square means; ^2^ grading backfat thickness: the average of two grading sites (BT between the last rib and first lumbar vertebra; BT between the 11th and 12th ribs); ^3^ intermuscular fat thickness: measured between the *latissimus dorsi* and *cutaneous trunci* muscles (at the 4th–5th thoracic vertebra cross-section).

**Table 3:** Porcine belly characteristics in groups categorized by VCS2000-meaured carcass weight and fat percentage.

Carcass Weight Measured by VCS2000 (W)	Heavy		Medium		Light		*p*-Value		
Fat Percentage Measured by VCS2000 (F)	High (*n* = 26)	Low (*n* = 8)	High (*n* = 24)	Low (*n* = 15)	High (*n* = 10)	Low (*n* = 17)	W	F	W × F
***VCS2000 measurements***									
Belly weight (kg)	13.3 ^a^ (0.12) ^1^	12.9 ^a^ (0.21)	12.3 ^b^ (0.12)	12.0 ^bc^ (0.15)	11.7 ^c^ (0.19)	11.3 ^c^ (0.14)	<0.001	<0.001	0.757
Fat weight of belly (kg)	5.06 ^a^ (0.12)	4.22 ^bc^ (0.21)	4.29 ^b^ (0.12)	3.88 ^c^ (0.15)	4.00 ^bc^ (0.19)	3.29 ^d^ (0.14)	<0.001	<0.001	0.323
Fat percentage of belly (%)	38.6 ^a^ (0.66)	33.4 ^b^ (1.19)	35.6 ^b^ (0.69)	31.8 ^c^ (0.87)	35.3 ^b^ (1.07)	30.2 ^c^ (0.82)	<0.001	<0.001	0.459
***Fat percentages of belly at various locations using image analysis***									
Fat percentage at 6th thoracic vertebra (%)	36.9 ^abCD^ (1.04)	32.7 ^bcBC^ (1.79)	37.6 ^aCD^ (1.11)	31.5 ^cC^ (1.46)	35.7 ^abD^ (1.60)	33.1 ^bcBC^ (1.41)	0.938	<0.001	0.402
Fat percentage at 10th thoracic vertebra (%)	44.5 ^abA^ (1.23)	39.3 ^bA^ (2.13)	45.3 ^aA^ (1.26)	40.9 ^abA^ (1.74)	44.3 ^abA^ (1.91)	41.4 ^abA^ (1.67)	0.766	0.003	0.806
Fat percentage at 11th thoracic vertebra (%)	41.8 ^abAB^ (1.28)	36.8 ^bAB^ (2.18)	42.7 ^aAB^ (1.28)	37.7 ^bAB^ (1.78)	42.3 ^abAB^ (1.95)	37.5 ^bAB^ (1.71)	0.884	<0.001	0.997
Fat percentage at 12th thoracic vertebra (%)	39.4 ^abBC^ (1.35)	34.1 ^bABC^ (2.34)	40.1 ^aBC^ (1.38)	34.8 ^bBC^ (1.91)	40.4 ^aBC^ (2.09)	35.5 ^abBC^ (1.83)	0.835	<0.001	0.987
Fat percentage at 13th thoracic vertebra (%)	37.9 ^aCD^ (1.28)	32.8 ^bBC^ (2.21)	38.2 ^aBCD^ (1.31)	33.4 ^bC^ (1.81)	38.0 ^aCD^ (1.98)	34.3 ^abBC^ (1.74)	0.899	0.002	0.921
Fat percentage at 14th thoracic vertebra (%)	35.6 ^aDE^ (1.12)	30.4 ^bC^ (1.94)	36.1 ^aCD^ (1.14)	31.5 ^bC^ (1.58)	35.5 ^abD^ (1.83)	31.1 ^bCD^ (1.52)	0.858	<0.001	0.958
Fat percentage at 4th lumbar vertebra (%)	32.5 ^aE^ (1.25)	30.1 ^abC^ (2.16)	33.7 ^aD^ (1.30)	31.1 ^abC^ (1.77)	30.4 ^abE^ (1.93)	27.3 ^bD^ (1.70)	0.125	0.050	0.970
***p*** **-Value**	<0.001	0.022	<0.001	<0.001	<0.001	<0.001			

^a–d^ Different superscripts in the same row represent significant differences (*p* < 0.05); ^A–E^ different superscripts in the same column represent significant differences (*p* < 0.05); ^1^ standard error of least-square means.

**Table 4:** Sensory evaluation of fat accumulation and appearance acceptability in belly cuts across groups categorized by VCS2000-measured carcass weight and fat percentage.

Carcass Weight Measured by VCS2000 (W)	Heavy		Medium		Light		*p*-Value		
Fat Percentage Measured by VCS2000 (F)	High (*n* = 26)	Low (*n* = 8)	High (*n* = 24)	Low (*n* = 15)	High (*n* = 10)	Low (*n* = 17)	W	F	W × F
***Degree of fat accumulation *** **^2^**									
Belly at 6th thoracic vertebra	3.21 ^aD^ (0.08) ^1^	2.60 ^bD^ (0.13)	2.43 ^bD^ (0.08)	2.61 ^bC^ (0.10)	2.43 ^bC^ (0.13)	2.60 ^bC^ (0.10)	<0.001	0.311	<0.001
Belly at 10th thoracic vertebra	4.77 ^aA^ (0.11)	3.95 ^bcA^ (0.17)	4.16 ^bcA^ (0.10)	3.78 ^cA^ (0.13)	4.14 ^bcA^ (0.17)	4.21 ^bA^ (0.13)	0.008	<0.001	0.009
Belly at 11th thoracic vertebra	4.37 ^aB^ (0.11)	3.43 ^cdB^ (0.17)	3.80 ^bcB^ (0.10)	2.93 ^eB^ (0.13)	3.98 ^bA^ (0.17)	3.28 ^dB^ (0.13)	<0.001	<0.001	0.682
Belly at 12th thoracic vertebra	3.65 ^aC^ (0.10)	2.98 ^cdC^ (0.15)	3.23 ^bcC^ (0.09)	2.31 ^eD^ (0.12)	3.38 ^abB^ (0.15)	2.78 ^dC^ (0.12)	<0.001	<0.001	0.340
Belly at 13th thoracic vertebra	3.11 ^aD^ (0.08)	2.29 ^cDE^ (0.13)	2.63 ^bD^ (0.08)	1.97 ^dE^ (0.10)	2.52 ^bcC^ (0.13)	2.25 ^cdD^ (0.10)	<0.001	<0.001	0.041
Belly at 14th thoracic vertebra	2.53 ^aE^ (0.07)	1.88 ^cdF^ (0.11)	2.15 ^bE^ (0.06)	1.44 ^eF^ (0.08)	2.02 ^bcD^ (0.11)	1.67 ^deE^ (0.08)	<0.001	<0.001	0.112
Belly at 4th lumbar vertebra	2.44 ^aE^ (0.06)	2.02 ^bEF^ (0.10)	1.98 ^bE^ (0.06)	2.00 ^bE^ (0.08)	1.64 ^cE^ (0.10)	2.04 ^bD^ (0.08)	<0.001	0.973	<0.001
***p*** **-** **V** **alue**	<0.001	<0.001	<0.001	<0.001	<0.001	<0.001			
***Appearance acceptability *** **^3^**									
Belly at 6th thoracic vertebra	6.16 ^dBC^ (0.13)	7.19 ^aB^ (0.20)	6.93 ^abcB^ (0.12)	6.68 ^bcB^ (0.15)	7.10 ^abB^ (0.20)	6.51 ^cdC^ (0.15)	0.645	0.615	<0.001
Belly at 10th thoracic vertebra	4.46 ^cE^ (0.13)	5.60 ^aE^ (0.20)	5.11 ^abE^ (0.12)	5.38 ^aD^ (0.16)	5.38 ^aE^ (0.20)	4.79 ^bcE^ (0.16)	0.352	0.045	<0.001
Belly at 11th thoracic vertebra	4.74 ^cE^ (0.13)	6.02 ^aE^ (0.20)	5.52 ^bD^ (0.12)	6.03 ^aC^ (0.15)	5.48 ^bE^ (0.20)	5.64 ^abD^ (0.15)	0.031	<0.001	0.003
Belly at 12th thoracic vertebra	5.69 ^dD^ (0.12)	6.69 ^abCD^ (0.19)	6.11 ^cdC^ (0.11)	6.78 ^aB^ (0.15)	6.05 ^cdD^ (0.19)	6.26 ^bcC^ (0.15)	0.090	<0.001	0.057
Belly at 13th thoracic vertebra	6.44 ^cB^ (0.10)	7.10 ^abBC^ (0.16)	6.98 ^bB^ (0.09)	7.43 ^aA^ (0.12)	7.12 ^abB^ (0.16)	6.90 ^bB^ (0.12)	0.002	0.004	0.004
Belly at 14th thoracic vertebra	6.94 ^bA^ (0.09)	7.76 ^aA^ (0.14)	7.43 ^aA^ (0.08)	7.71 ^aA^ (0.11)	7.69 ^aA^ (0.14)	7.54 ^aA^ (0.11)	0.057	<0.001	<0.001
Belly at 4th lumbar vertebra	5.87 ^cCD^ (0.09)	6.55 ^aD^ (0.14)	6.27 ^abC^ (0.08)	6.07 ^bcC^ (0.10)	6.50 ^aC^ (0.14)	5.82 ^cD^ (0.10)	0.892	0.449	<0.001
***p*** **-** **V** **alue**	<0.001	<0.001	<0.001	<0.001	<0.001	<0.001			

Each LSM was based on evaluations by 10 panelists for each carcass (n) within the respective group. ^a–e^ Different superscripts in the same row represent significant differences (*p* < 0.05); ^A–F^ different superscripts in the same column represent significant differences (*p* < 0.05); ^1^ standard error of least-square means; ^2^ score distribution: low to high, degree of fat accumulation: not at all to extremely high; ^3^ overall appearance acceptability: dislike extremely to like extremely.

## Data Availability

The original contributions presented in the study are included in the article. Further inquiries can be directed to the corresponding author.

## Abbreviations

The following abbreviations are used in this manuscript:

BT	Backfat thickness
GLM	General linear model
IFT	Intermuscular fat thickness
KAPE	Korea Institute of Animal Products Quality Evaluation
LEA	Loin eye area
LSM	Least squares mean
LV	Lumbar vertebra
PCA	Principal component analysis
SE	Standard error
TV	Thoracic vertebra
VBFP	Belly fat percentage measured by VCS2000
VBFW	Belly fat weight measured by VCS2000
VBW	Belly weight measured by VCS2000
VCW	Carcass weight measured by VCS2000
VFP	Total fat percentage measured by VCS2000
VFW	Total fat weight measured by VCS2000
VLP	Total lean meat percentage measured by VCS2000
VLW	Total lean meat weight measured by VCS2000
